# Current attitude of rheumatology practitioners on transition and transfer from pediatric to adult health care

**DOI:** 10.1186/1546-0096-9-S1-P274

**Published:** 2011-09-14

**Authors:** Deborah Hilderson, Philip Moons, Rene Westhovens, Carine Wouters

**Affiliations:** 1 Center for Health Services and Nursing Research, Katholieke Universiteit Leuven, Leuven, Belgium; 2 Division of Pediatrics, University Hospitals Leuven, Leuven, Belgium; 3 Rheumatology, Department of Musculoskeletal Sciences, University Hospitals Leuven, Leuven, Belgium; 4 Pediatric Rheumatology, University Hospitals Leuven, Leuven, Belgium

## Background

When patients with a rheumatic disorder reach adulthood, transfer to adult-focused care is advocated (1). A successful continuation of rheumatology follow-up beyond childhood depends on how clinicians think about transfer and transition (2).

## Aim

To explore the attitudes of rheumatology practitioners on transfer and transition from pediatric to adult health care of adolescents with a rheumatic disorder.

## Method

A survey (validated QUARTT, QUestionnaire about Attitudes of Rheumatology practitioners towards Transfer and Transition) among rheumatology practitioners attending the Pediatric Rheumatology European Society congress in 2010. Overall, 138 individuals participated, of which 59 were medical doctors (MDs) with paediatric and rheumatology background; 30 MDs with pediatric background; and 21 MDs with rheumatology background.

## Results

Participants judged that, when patients with active rheumatic disorder reach adulthood, they should receive medical follow-up from an adult rheumatologist (87%). Only 19% thought that patients should stay under surveillance of a paediatric rheumatologist. Several initiating factors for transfer were marked as important: readiness of the patient according to the caregiver (62%), age (61%) and psychosocial maturity (49%). A transfer meeting with the patient (76%), a referral letter (73%), or a medical transfer file (64%) were most preferred transfer communication tools with adult healthcare. Joint outpatient clinic, phone calls, and transfer meetings without the patient were considered to be less useful. The pediatric (94%) or adult rheumatologist (83%), parents (81%) and the nurse specialist (74%) were stated as most important active participants in the transition process.

## Conclusion

This study emphasized the importance of transfer to specialized rheumatology care of adolescents with an active rheumatic disease and highlighted transfer initiators and transfer communication tools.

